# Dosimetry and acute radiation enteritis comparison between prone and supine position in IMRT for gynecological cancers

**DOI:** 10.1002/acm2.14135

**Published:** 2023-08-24

**Authors:** Huamei Yan, Manya Wu, Wan Wang, Donghui Wang, Xiaoqing Huang, Jie Dong, Luxi Chen, Zhenghuan Li, Xiangying Xu

**Affiliations:** ^1^ Department of Radiation Oncology The Third Affiliated Hospital of Sun Yat‐Sen University Guangzhou China

**Keywords:** acute radiation enteritis, gynecologic cancers, Intensity‐modulated radiation therapy, positioning

## Abstract

**Purpose:**

To probe the differences of dosimetry and acute radiation enteritis between prone and supine position in gynecological cancer patients treated with intensity‐modulate radiotherapy (IMRT).

**Methods:**

Gynecologic tumor patients who received IMRT from January 2020 to July 2021 were analyzed. 60 patients were enrolled and divided into the supine or prone position group according to different radiotherapy positions, including 34 patients in prone position and 26 patients in supine position. The dose‐volume histogram of organs at risk (OARs) and the incidence of acute radiation enteritis were compared between the two groups. Multivariate logistic regression analysis was conducted to show the clinical characteristics and dose volume metrics to the association of acute radiation enteritis.

**Results:**

The percentage of volume receiving 5 Gy, 10 Gy, 15 Gy, 20 Gy, 30 Gy, 40 Gy, and 45 Gy doses for the small intestine were 79.0%, 67.4%, 59.6%, 44.3%, 17.0%, 8.9%, and 6.0%, respectively in the prone group, which were lower than those in the supine group (*P* < 0.05). The mean radiation dose (D_mean_) of the small intestine exposure in prone group was decreased (*P* < 0.001). Compared with the supine group, the prone group who suffered from acute radiation enteritis were much less. The probability of indigestion, nausea, vomiting, diarrhea, and abdominal pain in the prone position were 35.29%, 29.41%, 17.65%, 38.24%, and 5.88%, respectively. The differences in indigestion, nausea, and diarrhea between the two groups were statistically significant (*P* = 0.012, *P* = 0.029, and *P* = 0.041). Multivariate logistic regression analysis was shown that prone position was found to be protective against indigestion (*P* = 0.002), nausea (*P* = 0.013), vomiting (*P* = 0.035), and abdominal pain (*P* = 0.021).

**Conclusion:**

Prone position in IMRT for gynecological cancers could significantly reduce radiation dose to the small bowel and colon, which would decrease the occurrence and severity of acute intestinal side effects possibly.

## 1 INTRODUCTION

Cervical cancer and endometrial cancer are common malignant tumors and the primary cause of death among women in developing countries. They were mainly treated with intensity‐modulated radiation therapy (IMRT), which was recommended by the National Cancer Network (NCCN) of the United States.^1^, ^2^ Radiotherapy can improve the local control rate and overall survival rate of patients with cervical cancer.^3^ Benefit by the rapid development of the associated technology and equipment, radiotherapy has entered the era of precision treatment. IMRT is a form of radiotherapy that can more accurately localize the tumor target area. By administrating a three‐dimensional dose distribution that is highly conformable to the target, the IMRT treatment plan minimizes the dose to the organ at risk (OARs) and reduces the side effects of radiotherapy,^4^, ^5^, ^6^ which has an obvious dosimetry advantage over traditional radiotherapy in protecting the OARs.^7^

In the process of administering radiotherapy for gynecological tumors, the small intestine, colon, rectum, and bladder within the radiation field are highlighted as important OARs. The risk of side effects of radiotherapy is significantly related to the radiation dose and volume.^8^, ^9^ Radiation enteritis is a common side effect of pelvic radiotherapy, and is one of the main factors affecting the quality of life. As one of the organs with the lowest radiation tolerance, the small intestine is the main dose‐limiting organ for gynecological tumor radiotherapy and the main organ that presents side effects from radiotherapy. The incidence of radiation enteritis after cervical cancer radiotherapy is approximately 30.0%.^10^ Therefore, an important clinical research topic is determining ways to optimize the radiotherapy process to improve its efficacy and reduce its side effects.

Some measures have been implemented to reduce intestinal injury from pelvic radiotherapy,^11^ such as changing the position of radiotherapy treatment, changing the fixation technique, bladder filling, intestinal emptying, and injecting colloids between the bladder and rectum to expand the distance between the target volume and the OARs, and image‐guided radiotherapy. A number of studies^12^, ^13^, ^14^, ^15^ have confirmed that the use of the prone position can reduce the incidence of intestinal injury from radiation in rectal cancer, but whether the supine position or the prone position should be used for radiotherapy in gynecological tumor patients remains controversial. Pinkawa et al.^16^ reported that the use of the prone position for patients with cervical cancer or endometrial cancer reduced the radiation dose to the bladder but increased the radiation dose to the rectum. Adli et al.^17^ reported that the use of the prone position in gynecological tumor radiotherapy reduced the small intestine radiation dose. Prone positioning and Belly board fixation in gynecological tumor radiotherapy were found to reduce the incidence of acute small intestine injury.^18^ However, these studies only compared the dose‐volume relationships of the OARs or the incidence of intestinal injury, but few studies have focused on the differences in side effects after radiotherapy in different positions.

In this study, 60 cervical cancer and endometrial cancer patients underwent IMRT, while placed in the supine or prone positions, were analyzed in terms of the differences in the dose‐volume delivered to the OARs and the treatment side effects resulting from the different treatment positions. The goal of this study was to explore the differences of dosimetry and acute radiation enteritis between prone and supine position in gynecological cancer patients treated with IMRT, and probe whether prone position could reduce the radiation dose to OARs and the incidence of side effects.

## 2 MATERIALS AND METHODS

### 2.1 Study design and patients

Gynecological cancer patients who underwent IMRT in our hospital between January 2020 and July 2021 were included. Magnetic resonance imaging (MRI), computed tomography (CT) or positron emission tomography (PET)‐CT examinations before radiotherapy were performed to confirm the extent of tumor invasion and exclude the presence of distant metastases. Inclusion criteria: ① 18−75 years old, non‐pregnant or lactating women; ② Cervical cancer or endometrial cancer diagnosed by pathology; ③ Indications for radiotherapy; ④ ECOG score ≤2; ⑤ The function of important organs such as heart, lungs, liver, kidneys, and bone marrow can tolerate radiotherapy. Exclusion criteria: ① Patients with other uncontrolled serious diseases at the same time, with obvious abnormal functions of important organs; ② Previously received pelvic radiotherapy; ③ External beam radiation dose exceeds 50 Gy. Sixty‐nine gynecological cancer patients underwent IMRT were identified as potential candidates initially. Nine patients did not meet inclusion and exclusion criteria. Ultimately, 60 patients with cervical or endometrial cancer were enrolled in this study. Those patients who received IMRT were randomly positioned in supine or prone position according to the patient's request, which had nothing to do with physician preference, or patient health condition, or some assessment from prior imaging.

### 2.2 Simulation and contouring

**FIGURE 1:**
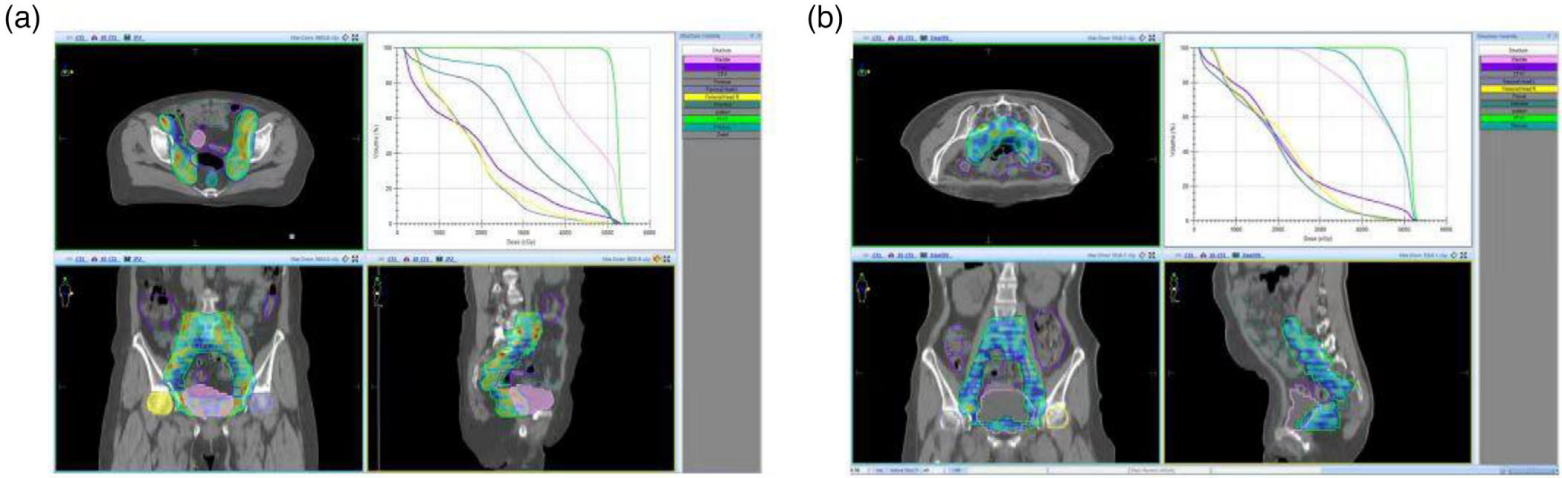
The distributions of the target volume and OARs in the prone and supine positions. (A) Supine position. (B) Prone position.

Patients in the prone position group were fixed with a Belly board and an Orfit frame combined with thermoplastic, and those in the supine position group were immobilized using a vacuum bag. All patients were asked to urinate one hour before the CT scan and drink 800 mL of water, then a bladder urine meter was used to measure the bladder volume, which was maintained between 200 and 300 mL. Patients were then scanned using a Siemens CT simulator (Simens, Somatom Definition, Germany) with a slice thickness of 5 mm. The scan ranged from the upper margin of the 11 thoracic vertebrae to 5 cm below the ischial tuberosity. Images were transmitted to a Monaco (V5.11, Elekta, Sweden) treatment planning system (TPS). The clinical tumor volume (CTV) included gross tumor (if present), sufficient vaginal area (at least 3 cm) from the gross tumor, parauterine and paravaginal soft tissues, pelvic lymphatic drainage area, and other lymphatic drainage areas (if necessary).^19^ The planning tumor volume (PTV) was obtained by adding a 5–10 mm margin to the CTV. The OARs included the small intestine, colon, rectum, bladder, femoral head, etc. CTV was delineated according to the consensus recommended by Lim et al.^20^ OARs were delineated according to the consensus recommended by Gay et al.^21^ The distribution of the target areas and OARs in the prone and supine positions were shown in Figure 1.

### 2.3 Treatment and planning

For all patients, 7‐field dynamic IMRT was designed using the Monaco TPS via the Monte Carlo (MC) algorithm, and IMRT treatment plans were delivered using an Elekta linear accelerator (Synergy, Elekta, weden) with x‐ray beam energy (6 MV). Target prescription dose: PTV 45–50 Gy/25 F, 1.8–2.0 Gy/F; 95% PTV was required to reach the prescribed dose, and no dose hot spots ≥ 110% appeared outside of the PTV. The max radiation dose (D_max_) of the small intestine was less than 50–52 Gy. The volume that received 45 Gy radiation dose (V_45_) was less than 100 cc, and V_30_ was less than 400 cc. The D_max_ of Colon was less than 52–55 Gy. The V_50_ of the bladder and rectum was both less than 50%, and femoral heads were less than 5%. The total dose 2cc of rectum, bowl, and bladder were less than 65–75 Gy, 70–75 Gy, and 80–90 Gy of brachy plus external beam radiotherapy. The bladder volume was measured before each treatment, and the difference between each treatment and during immobilization was controlled within 30%. Image‐guided radiotherapy was performed at least once a week by a cone‐beam computed tomography (CBCT) device (Elekta, Sweden), and the error of each treatment was kept within 5 mm.

### 2.4 Outcomes and assessments

The dose‐volume relationships of the OARs were analyzed, and the incidence and severity of acute radiation enteritis within 3 months after radiotherapy were recorded. The side effects were diagnosed and evaluated according to the National Institutes of Health (NIH) Common Adverse Events Evaluation Standard (CTCAE) 5.0. Grade 0; no AE. Grade 1 Mild; asymptomatic or mild symptoms; clinical or diagnostic observations only; intervention not indicated. Grade 2 Moderate; minimal, local or noninvasive intervention indicated; limiting age‐appropriate instrumental Activities of Daily Living (ADL). Grade 3 Severe or medically significant but not immediately life‐threatening; hospitalization or prolongation of hospitalization indicated; disabling; limiting selfcare ADL. Grade 4 Life‐threatening consequences; urgent intervention indicated. Grade 5; Death related to AE.

### 2.5 Statistical analysis

Quantitative data with a normal distribution are expressed as the mean ± standard deviation (SD), and a *t* test was used for comparisons between groups; for data with a skewed distribution, the median M (P25, P75) is presented, and the rank sum test (Wilcoxon Mann‐Whitney test) was used for comparisons between groups. Qualitative data were expressed as percentages, among which the Fisher exact probability method or chi‐square test was used for the comparison of disordered classification data, and the rank sum test was used for the comparison of ordered classification data. A *P* value less than 0.05 was considered to indicate a significant difference. Multivariate logistic regression analysis was conducted to show the clinical characteristics and dose volume metrics to the association of acute radiation enteritis. The rank correlation analysis was used to examine the association between these variables in participants with and without acute radiation enteritis. Prior to inclusion in the multivariate model, it was also necessary to test the continuous predictors for linearity in the logit, to ascertain whether the variables should be included as continuous or categorical. Variables with a significance probability of *P* ≤ 0.20 were then included in the multivariate logistic regression analysis, and non‐significant variables were removed in a backward stepwise elimination to determine the factors (*P* < 0.05) associated with acute radiation enteritis. The final models were tested for goodness of fit using the Hosmer and Lemeshow goodness‐of‐fit test. For this test, if the value of the chi square statistic in this test is low, the *P* value is not significant and indicates that the model is a good fit for the data. All statistical analysis was performed using SPSS 25.0 software.

## 3 RESULTS

### 3.1 Clinical characteristics

All of the 60 patients were non‐smokers. Twenty‐six patients in the supine position group consisted of 16 patients with cervical cancer and 10 patients with endometrial cancer. According to FIGO 2018 staging, the supine group included 7 patients in stage I, 9 patients in stage II, and 10 patients in stage III. The average age was 54 years (ranged 34–75 years), and the average of CTV was 573.69 cc (ranged 291.17–1720.21 cc). Thirty‐four patients in the prone position group consisted of 24 patients with cervical cancer and 10 patients with endometrial cancer, which included 5 patients in stage I, 12 patients in stage II, and 17 patients in stage III. The average age was 53.82 years (ranged 34–74 years), and the average of CTV was 578.13 cc (ranged 294.18–1032.09 cc). All the patients without surgery received brachytherapy, including seven patients in the supine group and eight patients in the prone group. The two groups were comparable in weight, body mass index (BMI), concurrent chemoradiotherapy (CCRT), previous surgery, brachytherapy, CTV and PTV. The baseline clinical characteristics of the two groups of patients were not significantly different, as shown in Table 1.

**TABLE 1:** Clinical characteristics of patients.

	Supine (n = 26)	Prone (n = 34)	*t*/*χ^2^/Z value*	*P*
**Age (year)**	54.00 ± 2.11	53.82 ± 1.75	0.07	0.949^a^
**Weight (kg)**	54.75 ± 2.07	54.98 ± 1.34	−0.10	0.921^a^
**BMI (kg/m^2^)**	22.62 ± 0.70	22.65 ± 0.51	−0.04	0.966^a^
**Diagnose**				
**Cervical cancer**	16 (40.00%)	24 (60.00%)	0.54	0.461^b^
**Endometrial cancer**	10 (50.00%)	10 (50.00%)		
**Stage**				
**I**	7 (26.92%)	5 (14.71%)	1.14	0.256^c^
**II**	9 (34.62%)	12 (35.29%)		
**III**	10 (38.46%)	17 (50.00%)		
**IV**	0 (0.00%)	0 (0.00%)		
**CCRT**				
**Yes**	8 (30.77%)	15 (44.12%)	1.11	0.292^b^
**No**	18 (69.23%)	19 (55.88%)		
**Surgery**				
**Yes**	19 (73.08%)	26 (76.47%)	0.09	0.764^b^
**No**	7 (26.92%)	8 (23.53%)		
**Brachytherapy**				
**Yes**	19 (73.08%)	26 (76.47%)	0.09	0.764^b^
**No**	7 (26.92%)	8 (23.53%)		
**CTV (cc)**	573.69 ± 40.94	578.13 ± 26.49	−0.10	0.925
**PTV (cc)**	968.36 ± 53.12	958.22 ± 31.37	0.17	0.863

*Note*: BMI, body mass index; CCRT, concurrent chemoradiotherapy; CTV, clinical tumor volume; PTV = planning tumor volume.

^a^ *t* test.

^b^ Chi‐square test.

^c^ Rank sum test.

### 3.2 Dose‐volume relationships of OARs

**FIGURE 2:**
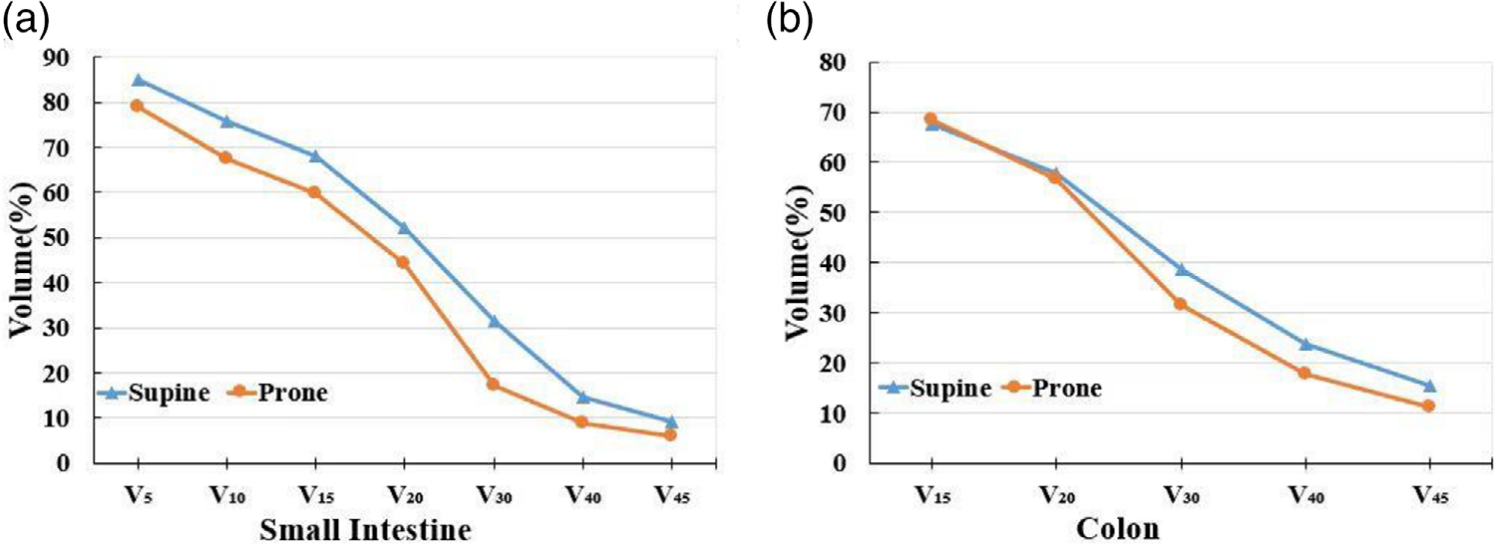
Volume relationships in different positions. (A) Small intestine. (B) Colon.

The radiation doses to the small intestine and a part of the colon in the prone group were lower than those in the supine group. The differences in V_5_, V_10_, V_15_, V_20_, V_30_, V_40_, and V_45_ of the small intestine were statistically significant (*P* = 0.014, *P* = 0.014, *P* = 0.017, *P* = 0.001, *P* < 0.001, *P* = 0.001 and *P* = 0.012, respectively), as shown in Table 2. The D_mean_ of the small intestine was also lower in the prone group than in the supine group (*P* < 0.001). The max radiation dose (D_max_) and V_50_ was not statistically significant in two groups (*P* > 0.05). However, the difference only in V_30_ and V_40_ of the colon was statistically significant (*P* = 0.032 and *P* = 0.031). The radiation doses to the bladder in the prone group were higher than those in the supine group. The dosimetry parameters of the rectum and femoral head were similar, as shown in Table 2 and Figure 2.

**TABLE 2:** Dose‐volume relationships of OARs in different positions.

	Supine(n = 26)	Prone(n = 34)	Difference and 95%CI	*t/Z value*	*P*
**Small Intestine**					
**V_5_ (%)**	84.92 (81.03, 93.19)	79.01 (73.77, 87.90)	6.58 (1.39–12.35)	‐2.46	0.014 ^b^
**V_10_ (%)**	75.86 ± 2.09	67.35 ± 2.45	8.50 (1.80–15.21)	2.54	0.014 ^a^
**V_15_ (%)**	67.91 ± 2.37	59.63 ± 2.32	8.28 (1.54–15.02)	2.46	0.017 ^a^
**V_20_ (%)**	52.16 (45.67, 66.23)	44.34 (37.65, 51.85)	11.26 (5.23–19.08)	‐3.48	0.001 ^b^
**V_30_ (%)**	31.29 (20.25, 42.97)	16.97 (13.49, 26.25)	13.02 (6.68–18.78)	‐4.25	<0.001 ^b^
**V_40_ (%)**	14.70 (10.37, 24.02)	8.89 (5.77, 13.63)	6.48 (3.15–10.68)	‐3.33	0.001 ^b^
**V_45_ (%)**	9.08 (5.97, 15.39)	6.01 (3.63, 9.98)	3.35 (0.93–6.66)	‐2.52	0.012 ^b^
**V_50_ (%)**	0.17 (0.00, 5.27)	1.67 (0.00, 4.70)	0.00 (−1.87–0.23)	‐0.71	0.476 ^b^
**D_mean_ (Gy)**	22.82 (20.36, 25.13)	18.69 (16.54, 22.17)	3.97 (1.91–6.01)	‐3.74	<0.001^b^
**D_max_ (Gy)**	50.48 (48.90, 53.14)	52.37 (48.81, 53.87)	‐0.45 (−2.03–0.54)	‐0.85	0.395 ^b^
**Colon**					
**V_15_ (%)**	67.48 ± 2.45	68.46 ± 1.34	‐0.97 (6.63–4.68)	‐0.35	0.730 ^a^
**V_20_ (%)**	57.8 ± 2.76	56.64 ± 1.72	1.16 (−5.39–7.71)	0.36	0.723 ^a^
**V_30_ (%)**	38.56 ± 2.65	31.39 ± 2.00	7.17 (0.65–13.69)	2.20	0.032 ^a^
**V_40_ (%)**	23.68 (19.40, 29.56)	17.77 (10.99, 25.89)	6.07 (0.34–11.06)	‐2.61	0.031 ^b^
**V_45_ (%)**	15.33 (11.66, 19.11)	11.14 (7.22, 18.02)	2.92 (−0.93–6.61)	‐1.43	0.154 ^b^
**V_50_ (%)**	0.16 (0.00, 9.06)	3.47 (0.00, 8.49)	0.00 (−2.95–0.29)	‐0.40	0.693 ^b^
**D_mean_ (Gy)**	24.68 ± 0.91	23.26 ± 0.64	1.42 (−0.73–3.57)	1.32	0.192 ^a^
**D_max_ (Gy)**	50.73 (48.88, 54.05)	53.66 (49.01, 54.50)	‐0.45 (−2.40–0.28)	‐1.16	0.245 ^b^
**Rectum**					
**V_15_ (%)**	100.00 (97.14, 100.00)	100.00 (99.17, 100.00)	0.00 (0.00–0.00)	‐0.80	0.426 ^b^
**V_20_ (%)**	100.00 (95.45, 100.00)	100.00 (98.30, 100.00)	0.00 (0.00–0.00)	‐0.09	0.926 ^b^
**V_30_ (%)**	94.90 (86.99, 100.00)	96.76 (92.73, 100.00)	‐0.62 (−4.76–0.82)	‐0.78	0.438 ^b^
**V_40_ (%)**	66.05 (47.96, 85.30)	74.84 (55.25, 85.00)	‐4.45 (−15.52–5.74)	‐0.87	0.387 ^b^
**V_45_ (%)**	44.86 ± 3.93	47.35 ± 2.81	‐2.48 (−11.89–6.93)	‐0.53	0.600 ^a^
**V_50_ (%)**	1.64 (0.00, 28.54)	15.00 (0.00, 34.75)	0.00 (−13.28–0.00)	‐1.13	0.259 ^b^
**D_mean_ (Gy)**	41.63 ± 0.71	42.89 ± 0.58	‐1.26 (−3.09–0.57)	‐1.38	0.174 ^a^
**D_max_ (Gy)**	50.43 (48.64, 50.09)	53.41 (49.08, 54.46)	‐0.47 (−2.05–0.28)	‐1.20	0.230 ^b^
**Bladder**					
**V_15_ (%)**	100.00 (98.92, 100.00)	100.00 (100.00, 100.00)	0.00 (−0.28–0.00)	‐2.53	0.011 ^b^
**V_20_ (%)**	99.13 (95.44, 100.00)	99.99 (99.71, 100.00)	‐0.42 (−2.59–0.00)	‐2.44	0.015 ^b^
**V_30_ (%)**	82.10 (73.30, 90.69)	94.90 (83.06, 98.79)	‐8.69 (−13.89–−3.09)	‐3.13	0.002 ^b^
**V_40_ (%)**	50.94 (43.23, 64.58)	68.66 (55.18, 73.89)	‐11.40 (−19.29–−4.91)	‐3.10	0.002 ^b^
**V_45_ (%)**	35.16 ± 2.31	44.17 ± 2.26	‐9.00 (−15.57–−2.44)	‐2.75	0.008 ^a^
**V_50_ (%)**	0.48 (0.00, 23.23)	25.53 (0.00, 33.33)	‐5.33 (−19.99–0.00)	‐2.02	0.044 ^b^
**D_mean_ (Gy)**	39.26 (36.64, 42.11)	42.72 (40.50, 44.49)	‐2.87 (−4.45–−1.20)	‐3.13	0.002 ^b^
**D_max_ (Gy)**	50.35 (49.06, 54.28)	53.80 (49.18, 54.38)	‐0.33 (−4.45–−1.20)	‐0.84	0.399 ^b^
**Femoral Head L**					
**V_30_ (%)**	16.36 ± 1.45	19.78 ± 1.25	‐3.43 (−7.25–0.39)	‐1.80	0.078 ^a^
**V_40_ (%)**	2.14 (0.76, 2.99)	3.12 (1.32, 5.53)	‐0.88 (−2.18–0.09)	‐1.82	0.069 ^b^
**V_50_ (%)**	0.00 (0.00, 0.00)	0.00 (0.00, 0.00)	0.00 (0.00–0.00)	‐0.22	0.828 ^b^
**D_mean_ (Gy)**	21.32 (17.99, 23.18)	21.85 (18.17, 23.12)	‐0.40 (−2.48–1.44)	‐0.48	0.633 ^b^
**D_max_ (Gy)**	46.03 (43.32, 48.59)	46.50 (44.88, 50.21)	‐1.09 (−3.02–0.96)	‐1.01	0.310 ^b^
**Femoral Head R**					
**V_30_ (%)**	16.66 ± 1.39	19.42 ± 1.18	‐2.76 (−6.40–0.87)	‐1.52	0.134 ^a^
**V_40_ (%)**	2.45 (0.87, 4.55)	2.78 (1.29, 5.33)	‐0.35 (−1.46–0.95)	‐0.43	0.665 ^b^
**V_50_ (%)**	0.00 (0.00, 0.00)	0.00 (0.00, 0.00)	0.00 (0.00–0.00)	‐0.50	0.614 ^b^
**D_mean_ (Gy)**	20.68 ± 0.78	20.50 ± 0.62	0.18 (−1.79–2.14)	0.18	0.857 ^a^
**D_max_ (Gy)**	46.50 ± 0.59	46.96 ± 0.53	‐0.47 (−2.07–1.13)	‐0.58	0.562 ^a^

^a^ *t* test.

^b^ Rank sum test.

### 3.3 Comparison of the incidence of acute radiation enteritis

**FIGURE 3:**
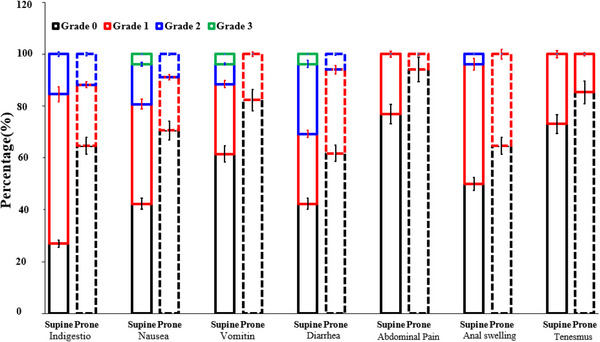
Incidence of side effects of patients with gynecological tumors treated with IMRT in prone or supine position.

Acute injury to the small intestine or colon caused by radiotherapy is mainly characterized by indigestion, nausea, vomiting, diarrhea, abdominal pain, etc. Acute rectal injury caused by radiotherapy mainly manifests as anal swelling, tenesmus, diarrhea, mucous stools, bloody stools, etc. The incidence of acute radiation enteritis in the two groups during radiotherapy and within 3 months after radiotherapy was analyzed. Compared with the supine group, the patients in the prone group who suffered from acute radiation enteritis were much less. The probability of indigestion, nausea, vomiting, diarrhea, and abdominal pain in the prone group contrast in the supine group were 35.29% versus 73.08%, 29.41% versus 57.69%, 17.65% versus 38.46%, 38.24% versus 57.69%, and 5.88% versus 23.08%, respectively. The probability of anal swelling, tenesmus, and mucous stools were 35.29% versus 50%, 14.71% versus 26.92%, and 0.00% versus 3.85%, respectively. The differences in indigestion, nausea, and diarrhea were statistically significant (*P* = 0.012, *P* = 0.029, *P* = 0.041). The incidence of anal swelling, tenesmus and mucous stools were similar in the two groups. No bloody stools occurred in these patients. No adverse reactions of grade 4 or above occurred, as shown in Table 3 and Figure 3. A few patients with grade 2 or above radiation enteritis received symptomatic treatment such as anti‐diarrhea and anti‐vomiting, but no patient stopped radiotherapy due to radiation enteritis.

**TABLE 3:** Acute radiation enteritis in the prone and supine positions in IMRT for gynecologic.

	Grade	Supine (n = 26)	Prone (n = 34)	*Z value*	*P*
**Indigestion**	0	7 (26.92%)	22 (64.71%)	‐2.507	0.012
	1	15 (57.69%)	8 (23.53%)		
	2	4 (15.38%)	4 (11.76%)		
	3	0 (0.00%)	0 (0.00%)		
	4‐5	0 (0.00%)	0 (0.00%)		
**Nausea**	0	11 (42.31%)	24 (70.59%)	‐2.18	0.029
	1	10 (38.46%)	7 (20.59%)		
	2	4 (15.38%)	3 (8.80%)		
	3	1 (3.85%)	0 (0.00%)		
	4‐5	0 (0.00%)	0 (0.00%)		
**Vomiting**	0	16 (61.54%)	28 (82.35%)	‐1.95	0.051
	1	7 (26.92%)	6 (17.65%)		
	2	2 (7.69%)	0 (0.00%)		
	3	1 (3.85%)	0 (0.00%)		
	4‐5	0 (0.00%)	0 (0.00%)		
**Diarrhea**	0	11 (42.31%)	21 (61.76%)	‐2.05	0.041
	1	7 (26.92%)	11 (32.35%)		
	2	7 (26.92%)	2 (5.88%)		
	3	1 (3.85%)	0 (0.00%)		
	4‐5	0 (0.00%)	0 (0.00%)		
**Abdominal pain**	0	20 (76.92%)	32 (94.12%)	‐1.93	0.054
	1	6 (23.08%)	2 (5.88%)		
	2	0 (0.00%)	0 (0.00%)		
	3	0 (0.00%)	0 (0.00%)		
	4‐5	0 (0.00%)	0 (0.00%)		
**Anal swelling**	0	13 (50.00%)	22 (64.71%)	‐1.23	0.218
	1	12 (46.15%)	12 (35.29%)		
	2	1 (3.85%)	0 (0.00%)		
	3	0 (0.00%)	0 (0.00%)		
	4‐5	0 (0.00%)	0 (0.00%)		
**Tenesmus**	0	19 (73.08%)	29 (85.29%)	‐1.16	0.245
	1	7 (26.92%)	5 (14.71%)		
	2	0 (0.00%)	0 (0.00%)		
	3	0 (0.00%)	0 (0.00%)		
	4‐5	0 (0.00%)	0 (0.00%)		
**Mucus stool**	0	25 (96.15%)	34 (100.00%)	‐1.14	0.253
	1	1 (3.85%)	0 (0.00%)		
	2	0 (0.00%)	0 (0.00%)		
	3	0 (0.00%)	0 (0.00%)		
	4‐5	0 (0.00%)	0 (0.00%)		
**Bloody stools**	0	26 (100.00%)	34 (100.00%)	0.00	1.000
	1	0 (0.00%)	0 (0.00%)		
	2	0 (0.00%)	0 (0.00%)		
	3	0 (0.00%)	0 (0.00%)		
	4‐5	0 (0.00%)	0 (0.00%)		

### 3.4 Association between variables and acute radiation enteritis

Overall, 51.67% (95% confidence interval [95% CI] = 38.65%–64.68%) of 60 participants reported that they had indigestion. The probability of nausea, vomiting, diarrhea, and abdominal pain were 41.67% (95% CI = 28.82%–54.51%), 26.67% (95% CI = 15.15%–38.19%), 46.67% (95% CI = 33.67%–59.66%), and 13.33% (95% CI = 4.48%–22.19%), respectively. The incidence of anal swelling, tenesmus, and mucous stools were 41.67% (95% CI = 28.82%–54.51%), 20.00% (95% CI = 9.58%–30.42%), and 1.67% (95% CI = −1.67%–5.00%), respectively. Rank correlation analysis was used to examine the association between variables in participants with and without acute radiation enteritis, which was presented in Table 4.

**TABLE 4:** Rank correlation analysis for association of variables with acute radiation enteritis.

	Indigestion		Nausea		Vomiting		Abdominal Pain		Diarrhea	
	*r value*	*P*	*r value*	*P*	*r value*	*P*	*r value*	*P*	*r value*	*P*
**Position**										
**Prone**	‐0.38	0.004	‐0.28	0.028	‐0.23	0.073	‐0.25	0.053	‐0.19	0.139
**Supine**	1.00		1.00		1.00		1.00		1.00	
**CCRT**										
**Yes**	0.21	0.101	0.31	0.017	0.46	0.000	0.20	0.136	0.16	0.235
**No**	1.00		1.00		1.00		1.00		1.00	
**Surgery**										
**No**	0.33	0.012	0.37	0.004	0.35	0.006	0.00	1.000	0.15	0.239
**Yes**	1.00		1.00		1.00		1.00		1.00	
**Brachytherapy**										
**Yes**	0.33	0.012	0.37	0.004	0.35	0.006	0.00	1.000	0.15	0.239
**No**	1.00		1.00		1.00		1.00		1.00	
**Stage**	0.13	0.326	0.04	0.755	0.07	0.574	0.26	0.048	0.01	0.962
**Age**	0.16	0.219	0.22	0.086	0.16	0.215	0.08	0.525	0.24	0.069
**BMI**	‐0.10	0.438	‐0.07	0.604	‐0.07	0.609	‐0.12	0.371	‐0.17	0.190
**CTV**	0.24	0.063	0.14	0.294	0.01	0.934	‐0.15	0.262	‐0.59	0.655
**PTV**	0.27	0.041	0.15	0.268	0.02	0.882	‐0.09	0.491	‐0.06	0.634
**Small intestine**										
**V_5_**	0.12	0.367	0.32	0.014	0.33	0.011	0.37	0.003	0.18	0.159
**V_10_**	0.05	0.688	0.28	0.028	0.23	0.073	0.33	0.009	0.14	0.290
**V_15_**	0.03	0.821	0.27	0.038	0.20	0.121	0.31	0.018	0.11	0.403
**V_20_**	0.12	0.360	0.20	0.125	0.18	0.162	0.24	0.064	0.17	0.195
**V_30_**	0.16	0.231	0.18	0.163	0.22	0.095	0.18	0.173	0.24	0.063
**V_40_**	0.10	0.438	0.20	0.123	0.22	0.087	0.11	0.413	0.24	0.060
**V_45_**	0.03	0.844	0.13	0.323	0.22	0.095	0.12	0.354	0.22	0.086
**D_mean_**	0.12	0.379	0.22	0.099	0.25	0.055	0.25	0.051	0.24	0.063
**Colon**										
**V_15_**	‐0.07	0.614	‐0.02	0.888	‐0.07	0.597	0.19	0.153	0.06	0.661
**D_mean_**	0.01	0.936	‐0.03	0.847	‐0.06	0.668	0.22	0.094	0.07	0.588

Abbreviations: CCRT, concurrent chemoradiotherapy; BMI, body mass index; CTV, clinical tumor volume; PTV,  planning tumor volume.

#### 3.4.1 Indigestion

Position (Prone), surgery, brachytherapy, and PTV were associated with indigestion (*P* < 0.05) (Table 4). All of the above variables, CCRT (*r* = 0.21, *P* = 0.101), and CTV (*r* = 0.24, *P* = 0.063) were initially included in the multivariate model. Position (Prone) and CCRT remained significantly associated with indigestion after multivariate analysis (Hosmer and Lemeshow goodness of fit, χ*^2^* = 4.04, *P* = 0.854), as shown in Table 5. Compared with supine position, prone position was found to be protective against indigestion (OR = 0.11, 95% CI = 0.03–0.44, *P* = 0.002). CCRT increased the risk of indigestion (OR = 4.77, 95% CI = 1.01–22.59, *P* = 0.049).

**TABLE 5:** Variables significantly associated with acute radiation enteritis following multivariate analysis.

	Indigestion		Nausea		Vomiting		Abdominal pain	
	OR (95% CI)	*P*	OR (95% CI)	*P*	OR (95% CI)	*P*	OR (95% CI)	*P*
**Position**								
**Prone**	0.11 (0.03–0.44)	0.002	0.19 (0.05–0.70)	0.013	0.01 (0.00–0.74)	0.035	0.10 (0.02–0.71)	0.021
**Supine**								
**CCRT**								
**Yes**	4.77 (1.01–22.59)	0.049	4.41 (1.10–17.68)	0.036	92.10 (3.88–2187.60)	0.005	3.80 (0.63–23.04)	0.147
**No**								

Abbreviations: OR, odds ratio; 95% CI, 95% confidence interval; CCRT,  concurrent chemoradiotherapy.

#### 3.4.2 Nausea

Position (Prone), CCRT, surgery, brachytherapy as well as V_5_, V_10_ and V_15_ of the small intestine were associated with nausea (*P* < 0.05) (Table 4). All of above the variables, Age (*r* = 0.22, *P* = 0.086), D_mean_ (*r* = 0.22, *P* = 0.099), V_20_ (*r* = 0.20, *P* = 0.125), V_30_ (*r* = 0.18, *P* = 0.163), and V_40_ (*r* = 0.20, *P* = 0.123) of the small intestine were initially included in the multivariate model. Position (Prone) and CCRT remained significantly associated with nausea after multivariate analysis (Hosmer and Lemeshow goodness of fit, *χ^2^* = 3.39, *P* = 0.908). Compared with supine position, prone position was found to be protective against nausea (OR = 0.19, 95% CI = 0.05–0.70, *P* = 0.013). CCRT increased the risk of nausea (OR = 4.41, 95% CI = 1.10–17.68, *P* = 0.036) (Table 5).

#### 3.4.3 Vomiting

CCRT, surgery, brachytherapy, and V_5_ of the small intestine were associated with vomiting (*P*< 0.05) (Table 4). All of the above variables, Position (Prone) (*r* = −0.23, *P* = 0.073), D_mean_ (*r* = 0.25, *P* = 0.055), V_10_ (*r* = 0.23, *P* = 0.073), V_15_ (*r* = 0.20, *P* = 0.121), V_20_ (*r* = 0.18, *P* = 0.162), V_30_ (*r* = 0.22, *P* = 0.095), V_40_ (*r* = 0.22, *P* = 0.087), and V_45_ (*r* = 0.22, *P* = 0.095) of the small intestine were initially included in the multivariate model. Position (Prone) and CCRT remained significantly associated with vomiting after multivariate analysis (Hosmer and Lemeshow goodness of fit, *χ^2^* = 7.71, *P* = 0.462). Compared with supine position, prone position was found to be protective against vomiting (OR = 0.01, 95% CI = 0.00–0.74, *P* = 0.035). CCRT increased the risk of vomiting (OR = 92.10, 95% CI = 3.88–2187.60, *P* = 0.005) (Table 5).

#### 3.4.4 Abdominal pain

V_5_, V_10_, and V_15_ of the small intestine and stage were associated with abdominal pain (*P*< 0.05) (Table 4). All of the above variables, Position (Prone) (*r* = −0.25, *P* = 0.053), CCRT (*r* = 0.20, *P* = 0.136), D_mean_ (*r* = 0.25, *P* = 0.051), V_20_ (*r* = 0.24, *P* = 0.064), and V_30_ (*r* = 0.18, *P* = 0.173) of the small intestine, as well as D_mean_ (*r* = 0.22, *P* = 0.094) and V_15_ (*r* = 0.19, *P* = 0.153) of the colon were initially included in the multivariate model. Position (Prone) remained significantly associated with abdominal pain after multivariate analysis (Hosmer and Lemeshow goodness of fit, *χ^2^* = 1.78, *P* = 0.971). Compared with supine position, prone position was found to be protective against abdominal pain (OR = 0.10, 95% CI = 0.02–0.71, *P* = 0.021). (Table 5).

No variables in participants associated with diarrhea was found by multivariate logistic regression analysis.

### 3.5 Predictors to grade 2 and above of acute radiation enteritis

Patients with grade 0 and grade 1 of acute radiation enteritis evaluated according to CTCAE were included in group 1, while patients with grade 2 and above were included in group 2. The differences between the two groups were analyzed by rank sum test or chi‐square test. Table 6 presented the variables with significant differences (*P* < 0.05). Patients with grade 2 and above of diarrhea were found that underwent radiotherapy in the supine position more than in prone position (*P* = 0.027). Patients with grade 2 and above of vomiting have a higher level of V_5_, V_10_, V_15_, V_20_, V_30_, and D_mean_ of the small intestine (*P* = 0.029, *P* = 0.026, *P* = 0.023, *P* = 0.020, *P* = 0.044, and *P* = 0.023, respectively) (Table 6). Similarly, patients with grade 2 and above of diarrhea have a higher level of V_5_, V_10_, V_15_, V_20_, V_30_, and D_mean_ of the small intestine (*P* = 0.011, *P* = 0.018, *P* = 0.043, *P* = 0.002, *P* = 0.028, and *P* = 0.049, respectively). A Lower level of BMI (*P* = 0.032) was observed in the patients with grade 2 and above of diarrhea. While, position for radiotherapy and dose‐volume relationships of the small intestine was similar in those patients with grade 2 and above of indigestion (expect V_5_ of the small intestine), nausea, and abdominal pain.

**TABLE 6:** Rank sum test for differences of variables with acute radiation enteritis.

	Grade 0 and 1	Grade 2 and above	Difference and 95%CI	*t/χ^2^/Z value*	*P*
**Indigestion**					
**Small intestine**					
**V_5_**	80.97 (74.87, 90.61)	91.22 (81.50, 98.71)	‐7.57 (−17.09–−0.72)	‐2.07	0.039
**Nausea**					
**Surgery**					
**Yes**	42 (93.33%)	3 (6.67%)		4.81	0.028
**No**	10 (66.67%)	5 (33.33%)			
**Brachytherapy**					
**Yes**	10 (66.67%)	5 (33.33%)		4.81	0.028
**No**	42 (93.33%)	3 (6.67%)			
**Vomiting**					
**Small intestine**					
**V_5_**	81.11 (74.89, 90.61)	96.80 (92.96, 98.07)	‐13.49 (−24.45–−2.15)	‐2.19	0.029
**V_10_**	70.80 (64.15, 79.61)	85.08 (82.85, 91.06)	‐15.85 (−31.17–−4.82)	‐2.22	0.026
**V_15_**	62.32 ± 13.15	80.27 ± 6.95	‐17.94 (−33.35–−2.54)	‐2.33	0.023
**V_20_**	46.26 (41.18, 53.12)	64.35 (62.59, 72.80)	‐19.60 (−36.81–−7.91)	‐2.32	0.020
**V_30_**	20.77 (16.07, 31.83)	48.32 (36.19, 59.13)	‐25.18 (−49.68–−0.12)	‐2.02	0.044
**D_mean_**	20.37 (18.34, 22.98)	29.09 (25.91, 32.22)	‐8.39 (−15.46–−1.24)	‐2.27	0.023
**Diarrhea**					
**Position**					
**Prone**	32 (94.12%)	2 (5.88%)		4.90	0.027
**Supine**	18 (69.23%)	8 (30.77%)			
**BMI**	22.68 (20.74, 24.61)	20.09 (18.74, 22.74)		‐2.14	0.032
**Small intestine**					
**V_5_**	80.81 (74.82, 90.61)	92.90 (83.50, 97.23)	‐8.80 (−16.62–−2.90)	‐2.56	0.011
**V_10_**	70.50 (62.78, 79.66)	78.15 (72.09, 90.21)	‐9.98 (−18.84–−1.35)	‐2.36	0.018
**V_15_**	61.89 (55.17, 67.36)	74.93 (61.74, 84.18)	‐11.22 (−20.76–−0.30)	‐2.02	0.043
**V_20_**	46.49 ± 12.57	61.88 ± 17.60	‐15.39 (−24.73–−6.04)	‐3.30	0.002
**V_30_**	20.23 (15.53, 32.08)	27.40 (22.56, 53.72)	‐8.32 (−18.78–−1.58)	‐2.20	0.028
**D_mean_**	20.15 ± 4.17	25.71 ± 7.63	‐5.56 (−11.09–−0.03)	‐2.24	0.049

Abbreviation: 95% CI,  95% confidence interval.

Variables with a significance probability of *P* ≤ 0.20 were then included in the multivariate logistic regression analysis. BMI, V_5_ and V_20_ of the small intestine were significantly associated with diarrhea after multivariate analysis (Hosmer and Lemeshow goodness of fit, *χ^2^* = 3.32, *P* = 0.912). BMI was found to be protective against diarrhea (OR = 0.49, 95% CI = 0.25–0.97, *P* = 0.041). V_5_ and V_20_ of the small intestine increased the risk of diarrhea (OR = 2.09, 95% CI = 1.01–4.32, *P* = 0.047, and OR = 2.90, 95% CI = 1.01–8.34, *P* = 0.048, respectively) (Table 7).

**TABLE 7:** Variables significantly associated with acute radiation enteritis following multivariate analysis.

	Diarrhea	
	OR(95% CI)	*P*
**BMI**	0.49 (0.25–0.97)	0.041
**Small intestine**		
**V_5_**	2.09 (1.01–4.32)	0.047
**V_20_**	2.90 (1.01–8.34)	0.048

Abbreviations: OR = odds ratio; 95% CI = 95% confidence interval; BMI = body mass index.

No variables in participants associated with grade 2 and above of indigestion, nausea, and vomiting were found by multivariate logistic regression analysis.

## 4 DISCUSSION

The subjects of this study were to analyze gynecological tumor patients who underwent radiotherapy in the supine or prone positions independently. It showed that the V_5_ to V_45_ of the small intestine for patients in the prone position was significantly lower than those in the supine position. The average dose of small intestine irradiation decreased from 22.82 Gy in the supine position to 18.69 Gy in the prone position (*P* < 0.001). The results of this study are basically consistent with reported values for small intestine irradiation of patients in the prone position.^22^, ^23^, ^24^ In addition, the V_20_ to V_45_ and D_mean_ of the colon in the prone position were lower than those in the supine position, but only the decreases in V_30_ and V_40_ were statistically significant. The reason may be that the small intestine as well as a part of colon fall into the abdominal hole of the Belly board due to gravity when the patient treated in the prone position, distancing the organ from the target field and thereby reducing the small intestine's irradiated volume and dose.

The incidence of acute radiation enteritis was lower in the prone group than in the supine group. The probability of indigestion, nausea, vomiting, diarrhea, and abdominal pain in the prone position were lower, and the differences in indigestion, nausea, and diarrhea were statistically significant (*P* = 0.012, *P* = 0.029, *P* = 0.041). The incidence and severity of acute radiation enteritis were reduced in the prone group, which may be due to patients in the prone position reducing the irradiated volume and dose of the small intestine as well as the colon, thereby reducing the amount of radiation intestinal injury.

Multivariate logistic regression analysis was conducted to show the clinical characteristics and dose volume metrics to the association of acute radiation enteritis. Compared with supine position, prone position was found to be protective against indigestion (OR = 0.11, 95% CI = 0.03–0.44, *P* = 0.002), nausea (OR = 0.19, 95% CI = 0.05–0.70, *P* = 0.013), vomiting (OR = 0.01, 95% CI = 0.00–0.74, *P* = 0.035), and abdominal pain (OR = 0.10, 95% CI = 0.02–0.71, *P* = 0.021). CCRT increased the risk of indigestion (OR = 4.77, 95% CI = 1.01–22.59, *P* = 0.049), nausea (OR = 4.41, 95% CI = 1.10–17.68, *P* = 0.036), and vomiting (OR = 92.10, 95% CI = 3.88–2187.60, *P* = 0.005). For grade 2 or above acute radiation enteritis, CCRT increased the risk of indigestion (OR = 6.32, 95% CI = 1.08–36.89, *P* = 0.040). V_5_ and V_20_ of the small intestine increased the risk of diarrhea (OR = 2.09, 95% CI = 1.01–4.32, *P* = 0.047, and OR = 2.90, 95% CI = 1.01–8.34, *P* = 0.048, respectively), BMI was found to be protective against diarrhea (OR = 0.49, 95% CI = 0.25–0.97, *P* = 0.041). Prone position, CCRT, BMI, V_5_, and V_20_ of the small intestine might be independent predictor of acute radiation enteritis.

It has been reported that the filling state of the bladder is related to the displacement of the anterior edge of the uterus, the volume of the bladder in the PTV, and the volume of the small intestine in the PTV.^25^, ^26^ Therefore, it is quite necessary to control the consistency of the bladder filling state for precise radiotherapy of gynecological tumors. In this study, the ultrasound meter used to measure the residual urine volume of the bladder was performed before each treatment to keep the degree of bladder filling basically the same and significantly weaken the influence of the degree of bladder filling on the small intestine radiation injury. There was no significant difference in the dose‐volume relationship of the rectum and femoral head between the two positions, which may be related to their relatively fixed anatomical structure, which is not easily changed regardless of patient position or degree of bladder filling.^27^

In summary, the use of the prone position in IMRT for gynecological malignant tumors can reduce the patient's small intestine radiation volume and dose as well as the incidence and severity of acute radiation enteritis. Due to age, physical condition and other factors, some patients may experience poor positioning, treatment discomfort, longer positioning times, poor postural repeatability, and increased positioning errors when placed in the prone position.^28^ Therefore, we suggest that immobilization with the prone position should be considered for IMRT in cooperative gynecological tumor patients.

## 5 CONCLUSIONS

In generally, IMRT for gynecologic cancer in the prone position has important clinical applicability, as it could significantly reduce the dose to the small intestine, which would possibly decrease the occurrence and severity of acute intestinal side effects.

## AUTHOR CONTRIBUTIONS

**Huamei Yan**: Funding acquisition, Writing Original Draft. **Manya Wu**: Conceptualization, Methodology. **Wan Wang**: Validation, Investigation. **Donghui Wang**: Formal analysis. **Xiaoqing Huang**: Resources. **Jie Dong**: Data Curation, Software. **Luxi Chen**: Visualization. **Zhenghuan Li**: Supervision, Writing Review & Editing. **Xiangying Xu**: Project administration, Writing Review & Editing.

## CONFLICT OF INTEREST STATEMENT

The authors have declared that there were no competing interests.

## ETHICS STATEMENT

The study was approved by the medical ethic committee of the Third Affiliated Hospital of Sun Yat‐sen University. The requirement for written informed consent was waived due to the retrospective nature of this study in line with the Ethical Guidelines for Medical and Health Research Involving Human Subjects in our country.

## Data Availability

The datasets used or analyzed during this current study are available from the corresponding author on reasonable request.
